# Evaluation of Effects of Chinese Prescription Kangen-karyu on Diabetes-Induced Alterations such as Oxidative Stress and Apoptosis in the Liver of Type 2 Diabetic *db/db* Mice

**DOI:** 10.1155/2012/143489

**Published:** 2012-08-30

**Authors:** Chan Hum Park, Jeong Sook Noh, Takuya Okamoto, Jong Cheol Park, Takako Yokozawa

**Affiliations:** ^1^Institute of Natural Medicine, University of Toyama, 2630 Sugitani, Toyama 930-0194, Japan; ^2^Iskra Industry Co., Ltd., 1-14-2 Nihonbashi, Chuo-ku, Tokyo 103-0027, Japan; ^3^Department of Oriental Medicine Resources, Sunchon National University, 315 Maegok-dong, Jeonnam 540-742, Republic of Korea; ^4^Organization for Promotion of Regional Collaboration, University of Toyama, 3190 Gofuku, Toyama 930-8555, Japan

## Abstract

The present study was conducted to examine whether Kangen-karyu has an ameliorative effect on diabetes-induced alterations such as oxidative stress and apoptosis in the liver of type 2 diabetic *db/db* mice. Kangen-karyu (100 or 200 mg/kg body weight/day, p.o.) was administered every day for 18 weeks to *db/db* mice and its effect was compared with vehicle-treated *db/db* and *m/m* mice. The administration of Kangen-karyu decreased the elevated serum glucose and leptin concentrations in *db/db* mice, and reduced the increased oxidative biomarkers including the generation of reactive oxygen species and lipid peroxidation in the liver. The *db/db* mice exhibited the upregulation of nicotinamide adenine dinucleotide phosphate oxidase subunits, NF-E2-related factor 2, heme oxygenase-1, nuclear factor-kappa B, cyclooxygenase-2, and inducible nitric oxide synthase levels in the liver; however, Kangen-karyu treatment significantly reduced those expressions. Moreover, the augmented expressions of apoptosis-related proteins, Bax, cytochrome *c*, c-Jun N-terminal kinase (JNK), phosphor-JNK, AP-1, and caspase-3, were downregulated by Kangen-karyu administration. Hematoxylin-eosin staining showed that the increased hepatocellular damage in the liver of *db/db* mice improved by Kangen-karyu administration. Our findings support the therapeutic evidence for Kangen-karyu ameliorating the development of diabetic hepatic complications *via* regulating oxidative stress and apoptosis.

## 1. Introduction

Diabetes mellitus is a major cause of mortality and morbidity worldwide, and its prevalence is increasing at an alarming rate. Type 2 diabetes is associated with nonalcoholic fatty liver disease (NAFLD), and estimates of the prevalence of NAFLD in this group have varied, with the highest being 70% [1]. This hepatic disorder, as a diabetic complication, is not only triggered by hyperglycemia-induced insulin resistance, but also by oxidative stress. Increased reactive oxygen species (ROS) generation activates stress-sensitive intracellular signaling pathways such as the transcription of nuclear factor-kappa B (NF-*κ*B), which plays a central role in inflammation-related disease [2]. Several researchers have demonstrated that ROS generation induced by nicotinamide adenine dinucleotide phosphate (NADPH) oxidase and the mitochondrial electron-transport chain is an early event in diabetic development [3, 4]. In addition, oxidative stress, in turn, activates different processes involving protein kinase C, cytokines, and others [2]. Several pathological responses have been suggested to induce diabetic complications such as hyperglycemia, oxidative stress-induced NF-*κ*B transcription, and apoptosis, which all take place synergistically and reciprocally. Therefore, novel approaches are necessary to identify therapeutic agents which can act with a pleiotropic effect including antioxidant properties to prevent and/or treat diabetic development.

Clinical evidence has suggested that the appropriate use of traditional Chinese medicines in conjunction with modern Western medicinal, or mainstream antidiabetic drugs, can prevent or ameliorate the development of diabetic complications. Many diabetic patients choose alternative therapeutic approaches such as herbal or traditional Chinese medicine along with the mainstream antidiabetic drugs, thus making alternative therapy for diabetes a popular remedy [5]. Among them, traditional Chinese prescriptions have received much attention as potential sources of novel therapeutic agents due to their multiple beneficial effects and absence of toxic and/or side effects [6].

Kangen-karyu (Guan-Yuan-Ke-Li in Chinese and has developed in Japan by the modification of herbal constituents of Kan-shin no. 2 (Guan-xin no. 2 in Chinese)) is a crude drug consisted of six herbs (Paeoniae Radix, Cnidii Rhizoma, Carthami Flos, Cyperi Rhizoma, Saussureae Radix, and Salviae Miltiorrhizae Radix) and has been clinically used as a treatment for cardiovascular diseases such as angina pectoris and cerebrovascular disorders [7]. Several studies demonstrated that Kangen-karyu exhibited biological activities such as platelet aggregation inhibition, hypertension suppression, the recovery of learning and memory impairment induced by senescence, neuroprotection, and an antidementia effect in animal experiments [8–11]. We have been conducting preclinical animal experiments related to diabetes using Kangen-karyu to examine its therapeutic potential. In our previous studies, we reported that Kangen-karyu showed beneficial effects on type 1 diabetes and its related complications through the suppression of protein expression related to advanced glycation endproducts [12]. The administration of Kangen-karyu to type 2 diabetic mice was suggested to make important contributions to prevent and/or delay the onset of diabetic hepatic disease [13–15]. To verify the effects of Kangen-karyu in the liver, the present study was undertaken to examine the efficacy of Kangen-karyu in the liver against oxidative stress and apoptosis brought about by hyperglycemia in type 2 diabetes.

## 2. Materials and Methods

### 2.1. Materials

Protease inhibitor mixture solution, 4,6-dihydroxy-2-mercaptopyrimidine (2-thiobarbituric acid, TBA), ethylenediaminetetraacetic acid (EDTA), and 10% neutral-buffered formalin were purchased from Wako Pure Chemical Industries, Ltd. (Osaka, Japan). 2′,7′-Dichlorofluorescein diacetate (DCFH-DA) was purchased from Molecular Probes (Eugene, OR, USA). The Bio-Rad protein assay kit and pure nitrocellulose membrane were purchased from Bio-Rad Laboratories (Tokyo, Japan). *β*-Actin, *o*-phthalaldehyde, and phenylmethylsulfonyl fluoride (PMSF) were purchased from Sigma Chemical Co. (St. Louis, MO, USA). Rabbit polyclonal antibodies against p22^phox^, NF-*κ*Bp65, nuclear factor erythroid 2-related factor 2 (Nrf-2), heme oxygenase-1 (HO-1), cytochrome *c*, Bax, and mouse monoclonal antibodies against cyclooxygenase-2 (COX-2), inducible nitric oxide synthase (iNOS), Bcl-2, and histone were purchased from Santa Cruz Biotechnology, Inc. (Santa Cruz, CA, USA). Rabbit polyclonal antibodies against c-Jun N-terminal kinase (JNK) and phosphor-JNK (p-JNK), and mouse monoclonal c-Jun were purchased from Cell Signaling Technology (Beverly, MA, USA). Rabbit polyclonal anti-reduced nicotinamide adenine dinucleotide phosphate oxidase-4 (Nox-4) (LifeSpan BioSciences, Seattle, WA, USA) and mouse monoclonal caspase-3 (BioVision Inc., Mountain View, CA, USA) were also used. Goat anti-rabbit and goat anti-mouse IgG horseradish peroxidase (HRP)-conjugated secondary antibodies were purchased from Santa Cruz Biotechnology, Inc. ECL Western Blotting Detection Reagents were purchased from GE Healthcare (Piscataway, NJ, USA).

### 2.2. Preparation of Kangen-karyu Extract

**Figure 1:**
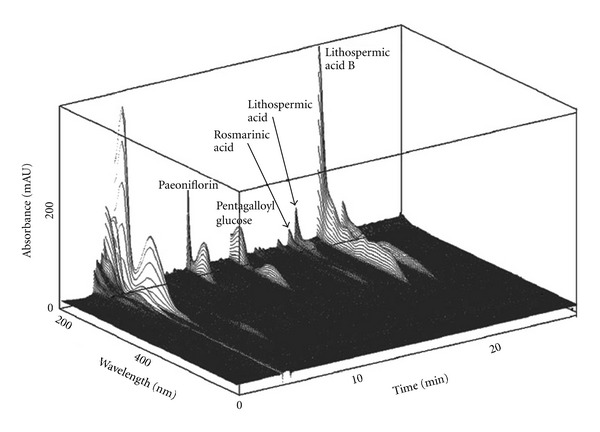
Three-dimensional HPLC analysis of Kangen-karyu showing its major compounds.

Kangen-karyu in the form of a dried powder extract was supplied by Iskra Co., Ltd. (Tokyo, Japan). The composition of Kangen-karyu used in this study was 2.25 g of Paeoniae Radix (*Paeonia lactiflora* Pallas root, Paeoniaceae), 2.25 g of Cnidii Rhizoma (*Cnidium officinale* Makino rhizome, Umbelliferae), 2.25 g of Carthami Flos (*Carthamus tinctrius* L. petal, Compositae), 1.125 g of Cyperi Rhizoma (*Cyperus rotundus* L. rhizome, Cyperaceae), 1.125 g of Aucklandiae Radix (*Aucklandia lappa* Dcne. root, Compositae), and 4.5 g of Salviae Miltiorrhizae Radix (*Salvia miltiorrhiza* Bunge root, Labiatae). This prescription was extracted with 25 volumes of water at 100°C for 1 h. After filtration, the solution was evaporated under reduced pressure to give an extract at a yield of 44%, by weight, of the starting materials. Each sample was dissolved in 50% aqueous ethanol with sonication, and filtered through a Cosmonice filter (PVDF, 0.45 *μ*m, Nacalai Tesque, Inc., Kyoto, Japan). Reverse-phase high-performance liquid chromatography was performed using a Cosmosil 5C_18_-AR II column (250 × 4.6 mm i.d., Nacalai Tesque, Inc.) with elution gradients of 4–30% (39 min) and 30–75% (15 min) CH_3_CN in 50 mM H_3_PO_4_ at a flow rate of 0.8 mL/min. The UV absorbance from 200 to 400 nm was monitored with a JASCO MD-910 photodiode array detector (Jasco, Tokyo, Japan). All assigned peaks were identified by carrying out co-injection tests with authentic samples and comparing with UV spectral data. Figure 1 shows the chromatogram obtained for Kangen-karyu. The major compounds detected were paeoniflorin, pentagalloyl glucose, rosmarinic acid, lithospermic acid, and lithospermic acid B. A voucher specimen has been deposited in the herbarium of the University of Toyama.

### 2.3. Experimental Animals and Treatment

 Animal experiments were performed according to the “Guidelines for Animal Experimentation” approved by the Ethics Committee of the University of Toyama (Registration no.: S-2006 INM-22). Six-week-old male C57BLKS/J *db/db* and their age-matched nondiabetic *m/m* mice were purchased from Japan SLC Inc. (Hamamatsu, Japan). Mice were maintained under a 12 h light/dark cycle and housed in a controlled temperature (23 ± 3°C) and humidity (about 60%). The mice were allowed free access to laboratory pellet chow (CLEA Japan Inc., Tokyo, Japan, comprising 24.0% protein, 3.5% lipids, and 60.5% carbohydrate) and water *ad libitum*. After adaptation (at 6 weeks of age), the glucose levels of blood taken from the tail vein were measured, and then *db/db* mice were divided into three groups (*n* = 8/group). Kangen-karyu (100 or 200 mg/kg body weight) was orally administered daily using a stomach tube, while vehicle-treated *db/db* mice were orally given water. The dose of Kangen-karyu used in this study was chosen based on the data obtained in our previous studies [13]. The nondiabetic *m/m* mice (*n* = 6) as a normal control group were used for comparisons with diabetic groups. The body weight, food intake, and water intake were measured every day during the administration period. After 18 weeks of administration, blood samples were collected by cardiac puncture from anesthetized mice. The serum was immediately separated from blood samples by centrifugation. Subsequently, mice were perfused with ice-cold physiological saline after cardiac puncture, and the liver was harvested, snap-frozen in liquid nitrogen, and stored at −80°C until analyses.

### 2.4. Analysis of Serum Parameters

 Serum glucose was measured using a commercial kit (Glucose CII-Test from Wako Pure Chemical Industries, Ltd., Osaka, Japan). The measurement of serum leptin and insulin (Morinaga Institute of Biological Science, Yokohama, Japan) was based on an enzyme-linked immunosorbent assay. Hepatic functional parameters (alanine aminotransferase, ALT and aspartate aminotransferase, AST) were measured using a Wako kit (Transaminase CII-Test).

### 2.5. Assessment of Hepatic ROS Generation and TBA-Reactive Substance (TBARS) Levels

ROS generation was measured employing the method of Ali et al. [16]. Hepatic tissues were homogenized on ice with 1 mM EDTA-50 mM sodium phosphate buffer (pH 7.4), and then 25 mM DCFH-DA was added to homogenates. After incubation for 30 min, the changes in fluorescence values were determined at an excitation wavelength of 486 nm and emission wavelength of 530 nm. The hepatic TBARS content was determined using the method of Uchiyama and Mihara [17].

### 2.6. Preparation of Nuclear and Postnuclear Fractions

 Nuclear protein extraction was performed according to the method of Komatsu [18]. In brief, hepatic tissues were homogenized with ice-cold lysis buffer containing 5 mM Tris-HCl (pH 7.5), 2 mM MgCl_2_, 15 mM CaCl_2_, and 1.5 M sucrose, and then 0.1 M dithiothreitol (DTT) and protease inhibitor cocktail were added. After centrifugation (10,500 xg for 20 min at 4°C), the pellet was suspended with extraction buffer containing 20 mM 2-[4-(2-hydroxyethyl)-1-piperazyl] ethanesulfonic acid (pH 7.9), 1.5 mM MgCl_2_, 0.42 M NaCl, 0.2 mM EDTA, and 25% (v/v) glycerol, and then 0.1 M DTT and protease inhibitor cocktail were added. The mixture was placed on ice for 30 min. The nuclear fraction was prepared by centrifugation at 20,500 xg for 5 min at 4°C.

The postnuclear fraction was extracted from the liver of each mouse as described below. In brief, hepatic tissue was homogenized with ice-cold lysis buffer (pH 7.4) containing 137 mM NaCl, 20 mM Tris-HCl, 1% Tween 20, 10% glycerol, 1 mM PMSF, and protease inhibitor cocktail. The homogenate was then centrifuged at 2,000 xg for 10 min at 4°C. The protein concentration in each fraction was determined using a Bio-Rad protein kit (Bio-Rad Laboratories, Hercules, CA, USA).

### 2.7. Immunoblotting Analyses

For the determination of Nrf2, NF-*κ*Bp65, AP-1, and histone, 15 *μ*g of protein from each nuclear fraction was electrophoresed through an 8% sodium dodecyl sulfate-polyacrylamide gel electrophoresis (SDS-PAGE). Separated proteins were transferred to a nitrocellulose membrane, blocked with 5% (w/v) skim milk solution for 1 h, and then incubated with primary antibodies to Nrf-2, NF-*κ*Bp65, AP-1, and histone, respectively, overnight at 4°C. After the blots were washed, they were incubated with anti-rabbit or anti-mouse IgG HRP-conjugated secondary antibody for 1.5 h at room temperature. Also, 15 *μ*g of protein of each postnuclear fraction of Nox-4, p22^phox^, HO-1, COX-2, iNOS, JNK, p-JNK, Bax, Bcl-2, cytochrome *c*, caspase-3, and *β*-actin was electrophoresed through 8–15% SDS-PAGE. Each antigen-antibody complex was visualized using ECL Western Blotting Detection Reagents and detected by chemiluminescence with LAS-4000 (Fujifilm, Tokyo, Japan). Band densities were determined using ATTO Densitograph Software (ATTO Corporation, Tokyo, Japan) and quantified as the ratio to histone or *β*-actin. The protein levels of groups are expressed relative to those of *m/m* mice (represented as 1).

### 2.8. Histological Examination

 The excised parts of livers were immediately fixed in 10% neutral-buffered formalin and, after embedding in paraffin, they were cut into 5-*μ*m-thick sections. After hematoxylin-eosin (HE) staining, these sections were examined with a light microscope.

### 2.9. Statistical Analysis

 Data are expressed as means ± S.E.M. Significance was assessed by one-way analysis of variance (ANOVA) followed by Dunnett's multiple comparison test (SPSS 11.5.1 for Windows, 2002, SPSS Inc., USA). Values of *P* < 0.05 were considered significant.

## 3. Results

### 3.1. General Characteristics

The food and water intakes as well as body weight of *db/db* mice after the 18-week experiment were significantly higher than those of the age-matched *m/m* mice. The administration of Kangen-karyu did not affect the food intake or body weight (data not shown). Also, they exhibited type 2 diabetic characteristics such as hyperglycemia, hyperleptinemia, and hyperinsulinemia compared with *m/m* mice, and Kangen-karyu administration significantly reduced serum glucose and leptin concentrations at a dose of 200 mg/kg. Regarding hepatic functional parameters, serum ALT and AST levels in vehicle *db/db* mice were elevated compared with those in *m/m* mice, while, in Kangen-karyu-administered *db/db* mice, these two parameters showed a slight decrease (Table 1).

### 3.2. Biomarkers Associated with Oxidative Stress in the Liver

As shown in Table 1, the levels of ROS and TBARS in the liver of vehicle-treated *db/db* mice were markedly higher than those of *m/m* mice, whereas these enhanced levels were significantly inhibited by the administration of both 100 and 200 mg/kg Kangen-karyu. The reduced ROS and TBARS levels in *db/db* vehicle mice recovered nearly to those of *m/m* mice in Kangen-karyu-treated groups.

### 3.3. Hepatic Oxidative Stress-Related Protein Expressions

**Figure 2:**
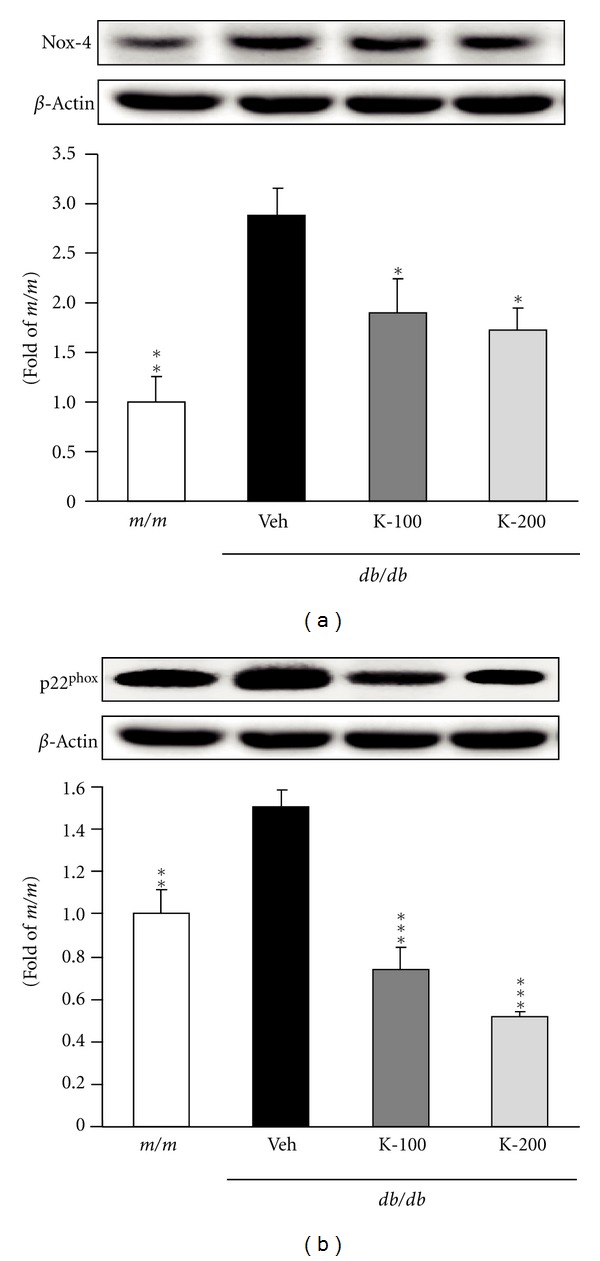
Nox-4 (a) and p22^phox^ (b) protein expressions in the liver. *m/m*: Misty; Veh: vehicle-treated *db/db* mice; K-100: Kangen-karyu 100 mg/kg body weight-treated *db/db* mice; K-200: Kangen-karyu 200 mg/kg body weight-treated *db/db* mice. Bars represent the means ± S.E.M. *P < 0.05, **P < 0.01, ***P < 0.001 versus vehicle-treated *db/db* mouse values.

**Figure 3:**
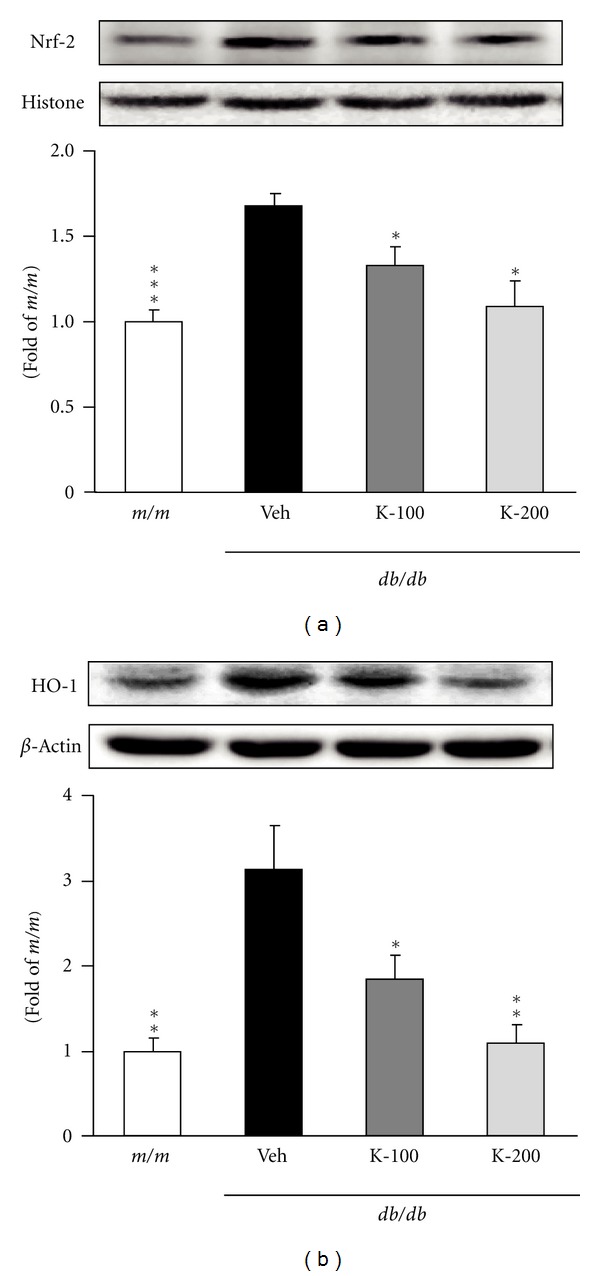
Nrf-2 (a) and HO-1 (b) protein expressions in the liver. *m/m*: Misty; Veh: vehicle-treated *db/db* mice; K-100: Kangen-karyu 100 mg/kg body weight-treated *db/db* mice; K-200: Kangen-karyu 200 mg/kg body weight-treated *db/db* mice. Bars represent the means ± S.E.M. *P < 0.05, **P < 0.01, ***P < 0.001 versus vehicle-treated *db/db* mouse values.

**Figure 4:**
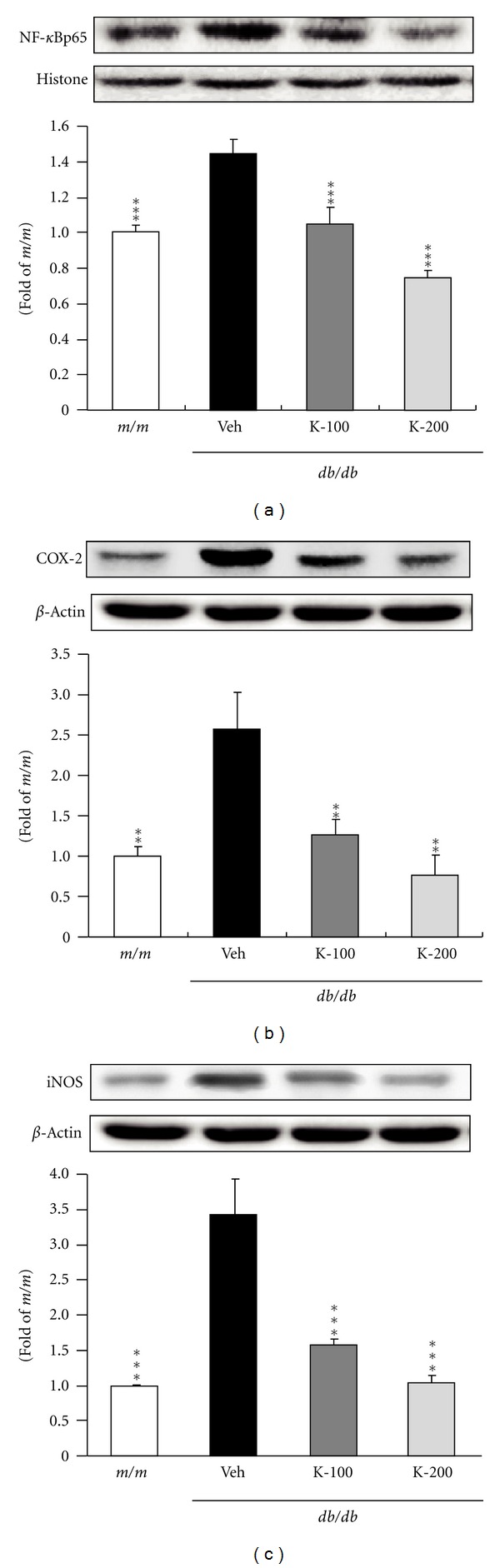
NF-*κ*Bp65 (a), COX-2 (b), and iNOS (c) protein expressions in the liver. *m/m*: Misty; Veh: vehicle-treated *db/db* mice; K-100: Kangen-karyu 100 mg/kg body weight-treated *db/db* mice; K-200: Kangen-karyu 200 mg/kg body weight-treated *db/db* mice. Bars represent the means ± S.E.M. *P < 0.05, **P < 0.01, ***P < 0.001 versus vehicle-treated *db/db* mouse values.

The effects of Kangen-karyu on hepatic Nox-4 and p22^phox^ protein expressions in *db/db* mice are shown in Figure 2. The expression level of hepatic Nox-4 in vehicle-treated *db/db* mice was markedly higher than that of *m/m* mice, whereas the groups administered Kangen-karyu showed a decreased expression compared with diabetic *db/db* mice, and there was a significant decrease in Nox-4 expression in both 100 and 200 mg/kg-treated mice (Figure 2(a)). In addition, compared with *m/m* mice, p22^phox^ expression was significantly upregulated in *db/db* control mice, but the oral administration of Kangen-karyu (100, 200 mg/kg body weight) led to significantly downregulated expression below the *m/m* value (Figure 2(b)). As shown in Figure 3, the protein expressions of Nrf-2 and HO-1 in vehicle-treated mice were significantly induced to a greater extent compared with *m/m* mice. In addition, each protein expression in Kangen-karyu-treated groups was notably decreased concentration-dependently (Figures 3(a) and 3(b)). Figure 4 shows the effect of Kangen-karyu administration on protein expressions of NF-*κ*Bp65, COX-2, and iNOS in hepatic tissue. All these proteins associated with oxidative stress showed significantly elevated expression in vehicle-treated *db/db* mice in comparison with *m/m* mice, while, in Kangen-karyu-administered *db/db* mice, these three parameters were significantly decreased (Figures 4(a), 4(b), and 4(c)).

### 3.4. Hepatic Apoptosis-Related Protein Expressions

**Figure 5:**
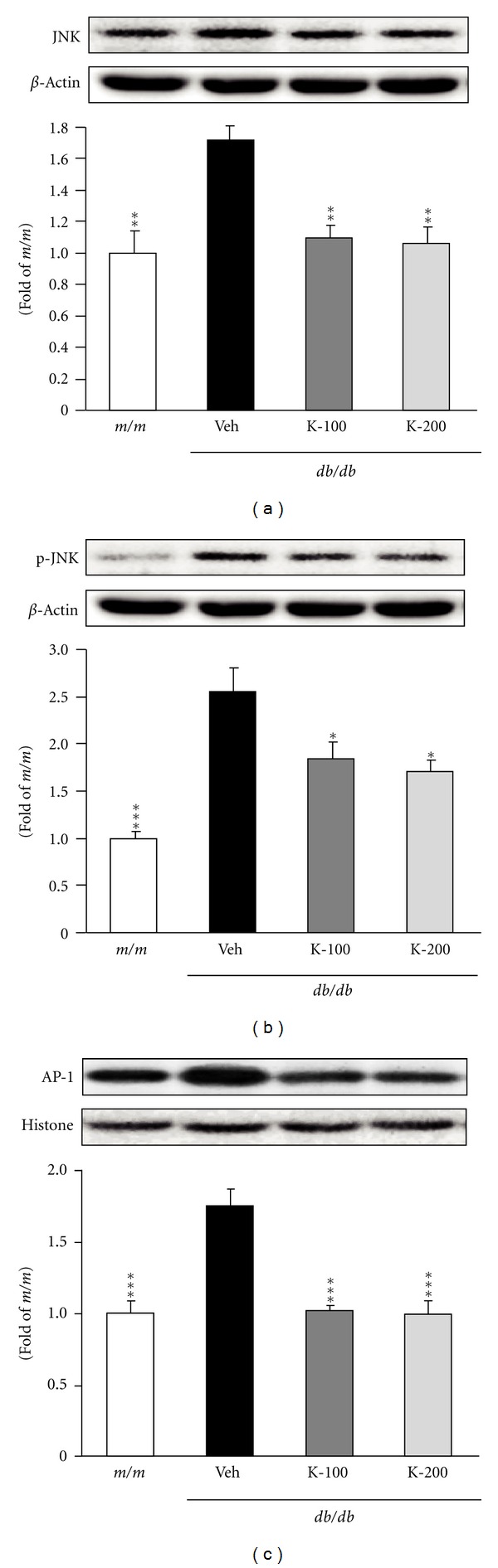
JNK (a), p-JNK (b), and AP-1 (c) protein expressions in the liver. *m/m*: Misty; Veh: vehicle-treated *db/db* mice; K-100: Kangen-karyu 100 mg/kg body weight-treated *db/db* mice; K-200: Kangen-karyu 200 mg/kg body weight-treated *db/db* mice. Bars represent the means ± S.E.M. *P < 0.05, **P < 0.01, ***P < 0.001 versus vehicle-treated *db/db* mouse values.

**Figure 6:**
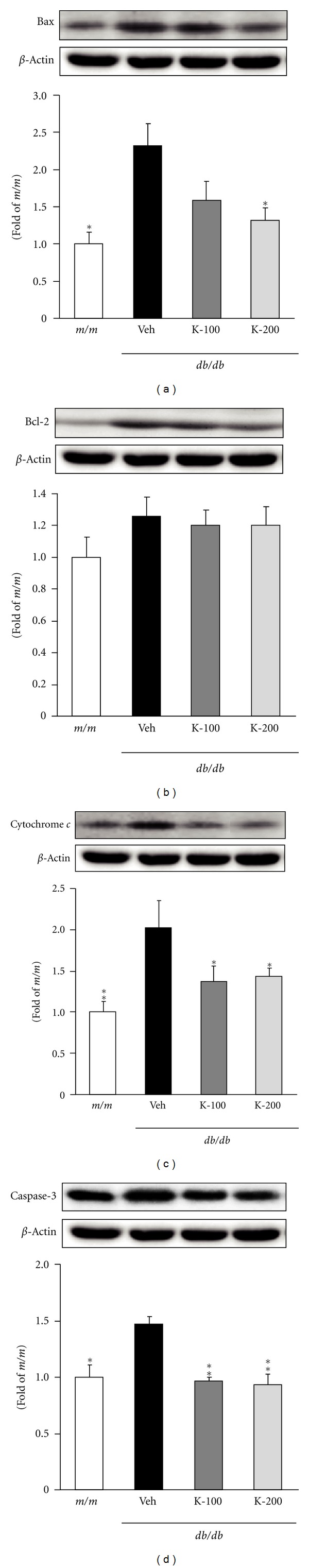
Bax (a), bcl-2 (b), cytochrome *c* (c), and caspase-3 (d) protein expressions in the liver. *m/m*: Misty; Veh: vehicle-treated *db/db* mice; K-100: Kangen-karyu 100 mg/kg body weight-treated *db/db* mice; K-200: Kangen-karyu 200 mg/kg body weight-treated *db/db* mice. Bars represent the means ± S.E.M. *P < 0.01, **P < 0.001 versus vehicle-treated *db/db* mouse values.

The expression levels of JNK, p-JNK, and AP-1 protein in the liver were analyzed (Figure 5). JNK, p-JNK, and AP-1 protein expressions in vehicle-treated *db/db* mice were significantly increased compared with those in *m/m* mice, whereas these enhanced levels were significantly decreased by the administration of both 100 and 200 mg/kg Kangen-karyu. In particular, the significant downregulation of JNK and AP-1 protein was observed.  Figure 6 shows the expressions of Bax, Bcl-2, cytochrome *c*, and caspase-3 in the liver. In the case of Bax protein, the vehicle-treated *db/db* mice showed elevated expressions compared with *m/m* mice, and groups administered Kangen-karyu showed decreased expressions concentration-dependently (Figure 6(a)). Cytochrome *c* and caspase-3 protein expressions were also higher in vehicle-treated *db/db* than *m/m* mice, but, in the 100 and 200 mg Kangen-karyu-administered group, these protein expressions were significantly decreased (Figures 6(c) and 6(d)). In contrast, Bcl-2 showed no changes in any experimental groups (Figure 6(b)).

### 3.5. Hepatic Histological Examination

**Figure 7:**
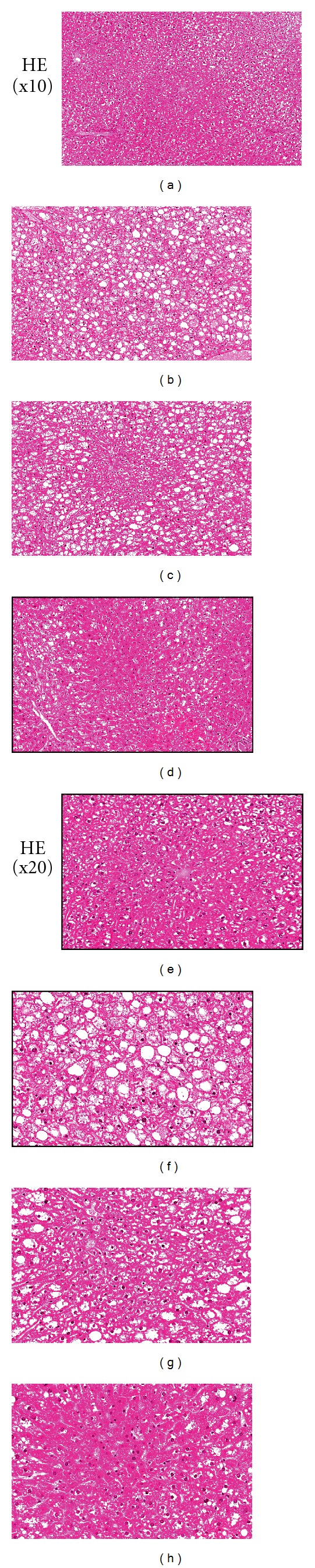
HE staining of hepatic tissue. Misty (a), vehicle-treated *db/db* mice (b), Kangen-karyu 100 mg/kg body weight-treated *db/db* mice (c), and Kangen-karyu 200 mg/kg body weight-treated *db/db* mice (d). X200.

Figure 7 shows the results of histological examinations using HE staining, which detects hepatocellular damage. The level of hepatocellular damage was higher in the liver of *db/db* vehicle mice compared with that of *m/m* mice. However, Kangen-karyu-administered *db/db* mice clearly showed decreased hepatocellular damage.

## 4. Discussion

In the human body, the liver is an important organ with various metabolic functions, and it plays a central role in whole-body lipid and carbohydrate metabolism *via* glycogen synthesis, lipogenesis, glycogenolysis, and gluconeogenesis [19]. Individuals with type 2 diabetes have a higher incidence of liver dysfunction than those without it [20]. Hyperglycemia largely depends on raised hepatic glucose production, and increased hepatic gluconeogenesis plays an important role in the pathophysiology [21]. Furthermore, fatty liver with type 2 diabetes is characterized by the accumulation of triglycerides within hepatocytes, and approximately 70% of persons with type 2 diabetes progresses toward this condition [1, 22]. Based on this evidence, dysfunction of the liver under type 2 diabetes can fully develop; therefore, we investigated the protective effect of Kangen-karyu in the liver against diabetic oxidative stress.

Mutations in the leptin receptor gene cause severe obesity and insulin resistance in rodents and humans. The cause of obesity in *db/db* mice is associated with a deficiency of leptin receptors depending on the genetic background, and these animals exhibit most of the human characteristics of type 2 diabetes including hyperglycemia in fasting and fed states, hyperinsulinemia, and insulin resistance [23, 24]. For this reason, *db/db* mice are valuable as an experimental model associated with type 2 diabetes. We administered Kangen-karyu for 18 weeks to diabetic *db/db* mice by oral injection and observed food intake following the administration. The administration of Kangen-karyu also slightly reduced both the weight gain and water intake of *db/db* mice. The administration of Kangen-karyu resulted in a significant decrease in levels of glucose and elevation of leptin in the serum, whereas it did not alter the serum insulin level. Lithospermic acid B, detected by HPLC analysis in Figure 1, has been reported to ameliorate the hyperglycemia through improving glucose tolerance in the type 2 diabetic OLETF rats [25]. Therefore, these results suggest that the hypoglycemic effect of Kangen-karyu might be partially derived from the augmentation of insulin sensitivity which is associated with insulin receptor substrate and glucose transporter protein expression.

 NADPH oxidase is a major source of ROS generation in hyperglycemia and is a multiunit enzyme that is activated during host defense in phagocytes. The activation of NADPH oxidase by stimulation leads to molecular oxygen and the production of superoxide. Furthermore, Nox-4, one of the NADPH oxidase multiple subunits, has been shown to produce hydrogen peroxide (H_2_O_2_) continuously, and the permeation of H_2_O_2_ may influence redox signaling. It is practicable to postulate that the regulation of Nox-4 transcription is an important step in modulating ROS production [26]. Then, Nox-4 and its regulatory partner p22^phox^ are responsible for the cellular responses to changes in oxygen levels [27]. According to these facts, we determined the expression of Nox-4 and p22^phox^ in hepatic tissue. Our data indicate that the expressions of both Nox-4 and p22^phox^ were decreased significantly by Kangen-karyu administration. This indicates that Kangen-karyu reduced free radicals, particularly superoxide and H_2_O_2_ in the liver, and consequently protected the liver from oxidative stress in the type 2 diabetic *db/db* mice model, which was confirmed from the results of attenuated oxidative stress including ROS and TBARS levels. Therefore, the administration of Kangen-karyu could attenuate the hepatic damage induced by oxidative stress in type 2 diabetes.

On the other hand, oxidative stress induces alterations in the Nrf2 complex, and its gene transcription, such as that of HO-1, is enhanced [28]. NADPH oxidase-derived ROS and the consequently induced activation of intracellular protein kinase cascades such as mitogen-activated protein kinase can mediate the induction of Nrf2 and HO-1 expressions [29, 30]. Besides ROS, advanced glycation endproducts, oxidized low-density lipoprotein, and transforming growth factor-*β*1 also activate Nrf2 transcription and increase HO-1 expression in various cell types [29–31]. Therefore, increased Nrf2-HO-1 pathway activation could be a biomarker of oxidative stress and an adaptive response under pathological conditions. In our results, type 2 diabetic *db/db* mice showed enhanced expressions of Nrf2 and HO-1 in the liver compared with normal *m/m* mice; however, Kangen-karyu treatment significantly reduced these expressions. These results suggest that Kangen-karyu administration effectively alleviates oxidative stress and results in the downregulation of Nrf2 and HO-1.

 NF-*κ*B is one of the most ubiquitous transcription factors and regulates the gene expression required for cellular proliferation, the inflammatory response, and cell adhesion [32]. Following inflammatory stimuli, excess NO by iNOS and proinflammatory prostaglandins by COX-2 have been reported to induce deleterious effects in the liver [33–35]. In this study, the increase in hepatic NF-*κ*B in *db/db *mice was downregulated by the administration of Kangen-karyu in a dose-dependent manner. These results were consequently related to inhibition of the expression of COX-2 and iNOS regulated by NF-*κ*B expression [36]. COX-2 and iNOS expressions were upregulated under insulin resistance, obesity, hyperglycemia, and oxidative stress; therefore, the downregulations of COX-2 and iNOS through the oral administration of Kangen-karyu demonstrated the inhibition of pathogenic factors in type 2 diabetes. Based on these results, Kangen-karyu has a crucial effect on the inflammation-activated signaling pathway through the regulation of NF-*κ*B, COX-2, and iNOS.

 ROS, generated by NADPH oxidase and/or mitochondrial metabolism, exert a significant effect on various redox-sensitive signaling processes, for example, cell growth, apoptosis, migration, and extracellular matrix modeling [37] and contribute to hepatocyte apoptosis [38]. Cell apoptosis induces cell death and, eventually, loss of function in tissues due to mitochondrial dysfunction including membrane potential loss, the upregulation of Bax, and release of cytochrome *c* [39]. It has been shown that Bax may influence permeability and the release of cytochrome *c* from inter-membrane spaces into the cytosol, while Bcl-2 is able to stabilize and inhibit membrane pore opening, ultimately protecting against oxidative stress [40]. Cytochrome *c* release from mitochondria is a critical step in the apoptotic cascade, and this activates downstream caspases such as caspase-3, which has been implicated in the pathogenesis of hepatic injury and may be blocked by antioxidants [41]. In this study, Kangen-karyu treatment of *db/db* mice significantly suppressed hepatic protein expressions of Bax, cytochrome *c*, and caspase-3, although there were no changes in Bcl-2 protein levels among all experimental groups. The results presented here suggest that Kangen-karyu could prevent apoptosis-induced hepatic damage, at least in part, through the amelioration of oxidative stress-induced mitochondrial dysfunction. 

On the other hand, the JNK/AP-1 signaling pathway acts as a multifunctional regulator of cellular proliferation and differentiation in many tissues. The role of JNK/AP-1 extends to the regulation of cell survival as it functions as an antiapoptotic promoter of tumorigenesis and stress-related inducer of programmed cell death [42, 43]. Studies on cell culture systems have established a critical role for JNK/AP-1 signaling in hepatocyte apoptosis, particularly that resulting from oxidant-induced injury [44]. In our Western blotting analysis, experimental type 2 diabetes resulted in the increased expressions of JNK/AP-1 proteins, whereas the expressions of these proteins were markedly reduced by Kangen-karyu administration. These results showed that the antiapoptotic effects of Kangen-karyu may be associated with the downregulation of apoptotic proteins in the liver of type 2 diabetic mice.

 Kangen-karyu had a pleiotropic effect on several oxidative stress-related parameters and exerted a hepatoprotective effect on the development of diabetic complications in type 2 diabetic *db/db* mice. Collectively, the present study provides important evidence that Kangen-karyu exerts its hepatoprotective potential mainly through its antioxidant properties during the development of diabetic complications in type 2 diabetes. Higher serum levels of ALT and AST were decreased in the Kangen-karyu-treated group. These results, concomitant with the effect on histological changes, indicate that Kangen-karyu protects against hepatic injury induced in type 2 diabetic *db/db* mice. However, differences in sensitivity to the effects of Kangen-karyu of several pathological responses (hyperglycemia, oxidative stress-induced NF-*κ*B transcription, and apoptosis) exist, and further studies on these differences may facilitate the characterization of more detailed therapeutic mechanisms of Kangen-karyu.

**Figure 8:**
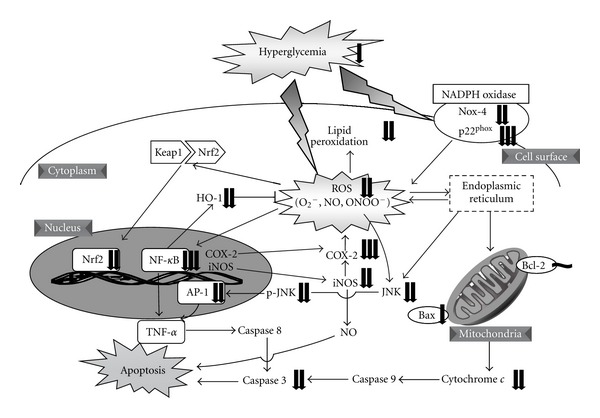
Predictable mechanisms in hepatic tissues administered Kangen-karyu against oxidative stress and apoptosis. The Kangen-karyu attenuated hyperglycemia and hyperleptinemia, and subsequently decreased ROS and TBARS production in the liver. Further, Kangen-karyu ameliorated the values of proinflammatory COX-2 and iNOS proteins regulated by NF-*κ*B, oxidative stress defense factor Nrf2 and HO-1 proteins, and apoptotic JNK, p-JNK, AP-1, Bax, and cytochrome *c *proteins.

In conclusion, this report demonstrated that the administration of Kangen-karyu could ameliorate hepatic dysfunction, especially due to oxidative stress and apoptosis, in type 2 diabetes, as summarized in Figure 8. These results might be supported by previous evidence of its constituents observed in the results of HPLC analysis, that is, lithospermic acid B showed hepatoprotective activity in liver injury [45], rosmarinic acid might ameliorate the lipid profile as a peroxisome proliferator-activated receptor *α* agonist [46], and paeoniflorin influenced lipid metabolism [47]; therefore, the present study importantly suggests that the administration of Kangen-karyu may become a new therapeutic strategy for hepatic dysfunction in type 2 diabetes.

## Figures and Tables

**Table 1:** Biochemical analyses.

Parameters	*m/m*	*db/db*		
		Veh	K-100	K-200
Serum glucose (mg/dL)	132.5 ± 4.9^∗∗∗^	487.1 ± 11.4	458.5 ± 24.4	423.5 ± 3.8^∗∗^
Serum leptin (ng/mL)	1.70 ± 0.22^∗∗∗^	20.36 ± 0.90	18.73 ± 1.19	15.18 ± 0.28^∗∗∗^
Serum insulin (ng/mL)	1.58 ± 0.03^∗∗∗^	3.26 ± 0.17	3.55 ± 0.34	3.74 ± 0.21
Serum ALT (IU/L)	35.0 ± 2.2^∗^	88.3 ± 14.3	79.6 ± 7.8	76.8 ± 12.3
Serum AST (IU/L)	10.6 ± 0.4^∗∗∗^	53.9 ± 3.8	48.4 ± 6.3	40.1 ± 7.0
Hepatic ROS (fluorescence/min/mg protein)	2129 ± 58^∗∗∗^	2906 ± 68	2594 ± 55^∗∗^	2158 ± 155^∗∗^
Hepatic TBARS (nmol/mg protein)	2.02 ± 0.11^∗∗^	2.98 ± 0.22	2.24 ± 0.17^∗^	1.47 ± 0.06^∗∗∗^

*m/m*: Misty; Veh: vehicle-treated *db/db* mice; K-100: Kangen-karyu 100 mg/kg body weight-treated *db/db* mice; K-200: Kangen-karyu 200 mg/kg body weight-treated *db/db* mice. The results are presented as the means ± S.E.M. ^∗^ *P* < 0.05, ^∗∗^ *P* < 0.01, ^∗∗∗^ *P* < 0.001 versus vehicle-treated *db/db* mouse values.
